# Nanoparticle-Reinforced Electroless Composite Coatings for Pipeline Steel: Synthesis and Characterization

**DOI:** 10.3390/ma18173949

**Published:** 2025-08-22

**Authors:** Biplab Baran Mandal, Vikash Kumar, Sovan Sahoo, Buddhadeb Oraon, Sumanta Mukherjee

**Affiliations:** 1 Department of Mechanical Engineering, Jadavpur University, Kolkata 700032, India; bbmandal.mech.rs@jadavpuruniversity.in (B.B.M.); vikashk.mech.rs@jadavpuruniversity.in (V.K.); sovans.mech.rs@jadavpuruniversity.in (S.S.); buddhadeb.oraon@jadavpuruniversity.in (B.O.); 2 Department of Production & Industrial Engineering, BIT Sindri, Dhanbad 828123, India

**Keywords:** pipeline steel, electroless coatings, composite coatings, heat treatment, hardness, wear

## Abstract

Protective coatings are essential for extending the service life of components exposed to harsh conditions, such as pipes used in industrial systems, where wear and corrosion remain constant challenges. This study explores the development of a nano-sized TiO_2_-reinforced electroless nickel-based ternary (Ni-W-P) alloy and composite coating on API X60 steel, a high-strength carbon steel pipe grade widely used in oil and gas pipelines, using an alkaline hypophosphite-reduced bath. The surface morphology, microstructure, elemental composition, structure, phase evolution, adhesion, and roughness of the coatings were analyzed using optical microscopy, FESEM, EDS, XRD, AFM, cross-cut tape test, and 3D profilometry. The tribological performance was evaluated via Vickers microhardness measurements and reciprocating wear tests conducted under dry conditions at a 5 N load. The TiO_2_ nanoparticle-reinforced composite coating achieved a consistent thickness of approximately 24 µm and exhibited enhanced microhardness and reduced coefficient of friction (COF), although the addition of nanoparticles increased surface roughness (S_a_). Annealing the electroless composites at 400 °C led to a significant improvement in their tribological properties, primarily owing to the grain growth, phase transformation, and Ni_3_P crystallization. XRD analysis revealed phase evolution from an amorphous state to crystalline Ni_3_P upon annealing. Both the alloy and composite coatings exhibited excellent adhesion performances. The combined effect of TiO_2_ nanoparticles, tungsten, and Ni_3_P crystallization greatly improved the wear resistance, with abrasive and adhesive wear identified as the dominant mechanisms, making these coatings well suited for high-wear applications.

## 1. Introduction

Pipelines and other critical components in industrial systems often operate under harsh service conditions, where severe wear and corrosion can significantly reduce their operational lifespans. For instance, API 5L X42, X52, X60, X65, X70, X80, etc., carbon–manganese steels are commonly used for oil and gas pipelines owing to their excellent mechanical properties; however, these materials remain vulnerable to surface degradation in aggressive environments [1,2]. To address these challenges and enhance the durability of such components, various surface engineering techniques have been developed, including electroplating [3], thermal spraying [4], physical and chemical vapor deposition [5], hardfacing [6], and laser surface treatment techniques [7,8]. Although these methods can effectively improve the surface hardness and corrosion resistance, they may involve complex equipment, high processing temperatures, or difficulties in achieving uniform coatings on intricate geometries.

In comparison, electroless (EL) coatings, also known as autocatalytic plating, are chemical deposition techniques that can produce uniform and adherent deposits on components with complex shapes without requiring any sophisticated equipment or external power sources, and, therefore, have attracted particular interest. This process relies on controlled chemical reduction to form precise metallic or non-metallic coatings, thereby overcoming some of the limitations associated with conventional electroplating methods [9,10]. EL coatings are generally classified into pure nickel, alloy, composite, and multi-alloy coatings, each offering specific performance advantages. The deposition process occurs in an aqueous bath, typically consisting of metal ions, reducing agents, complexing agents, pH adjusters, and stabilizers. The bath composition plays an essential role in determining not only the properties of the final coating, but also the overall efficiency of the coating process [11,12,13,14]. During the coating process, nickel (Ni) is deposited through a chemical reduction process in an aqueous medium, facilitated by a reductant that donates electrons to convert nickel ions into their metallic state [15,16]; therefore, these coatings are categorized by the type of reductant or reducing agent. For instance, hypophosphite baths are generally used to deposit electroless nickel–phosphorous (Ni-P) coatings, whereas hydrazine- and borohydride-based baths reduce pure nickel and nickel-boron (Ni-B) coatings [17]. Multi-alloy, poly-alloy, or ternary electroless coatings are produced by co-depositing a third metal, such as Co, W, Cu, Mn, Mo, Zn, Re, or Ce, with binary Ni-P or Ni-B matrices [18]. The addition of tungsten to EL alloy coatings has attracted substantial interest because of its exceptional hardness, improved wear, and corrosion protection [19,20,21,22,23]. In contrast, tungsten-based ternary alloy coatings are considered potential substitutes for hard-chrome coatings [24].

The inclusion of hard or soft ceramic particles in binary and ternary alloy coatings has led to substantial progress in electroless (EL) coatings, enhancing their mechanical properties, wear resistance, and overall functional performance [21,23]. Furthermore, incorporating nano-sized particles into multi-alloy or alloy coatings produces nanoparticle-reinforced nickel-based EL composite coatings, providing a synergistic strategy to enhance the overall coating properties [22,25,26,27]. Composite coatings can serve different purposes in various sectors such as automobiles, electronics, and petroleum [28,29]. Incorporating nanomaterials into the ternary EL nickel alloy matrix not only modifies the morphology and structure of the coating, but also enhances the chemical and mechanical properties of the EL deposits [30,31,32,33,34]. A few researchers have focused on the effect of incorporating nanoparticles into EL ternary Ni-W-P alloy coatings. Ranganatha et al. [35] introduced nano-sized WS_2_ and MoS_2_ into Ni-W-P coatings, resulting in composite coatings that reduced the friction coefficient and made them corrosion-resistant. The inclusion of these nanoparticles had little or no noticeable effect on the microhardness of the EL composite films. Alternatively, the inclusion of nanoparticles enhances the deposit uniformity and results in a more refined grain structure in the coatings. Miri et al. [36] demonstrated that incorporating Montmorillonite (MMT) nanoparticles into Ni-B coatings enhances microhardness and corrosion resistance, reducing the *i*_corr_ value 0.12 to 0.03 μA/cm^2^ due to the nanoparticles’ dispersion strengthening and improved barrier properties. Silicon Carbide (SiC) increased the microhardness of Ni-W coatings by inhibiting grain growth, obstructing dislocation movement, and enhancing the composite structure through dispersion hardening [37]; the peak microhardness attained was 970 HV_0.05_, which is a result of the strengthening mechanism triggered by SiC particles during electroless deposition. By preventing the formation of delicate intermetallic phases, Bae et al. [38] demonstrated that the application of a Ni-W-P coating as a protective layer significantly improved the thermal stability and adhesion strength of solder junctions in Bi_2_Te_3_ thermo-electric modules. The inclusion of ZnO and SiC nanoparticles enhanced the microhardness of the EL composite coatings by augmenting the load-bearing capability, promoting even particle distribution, and facilitating the formation of a hard crystalline Ni_3_P phase during annealing [39]. Yi He et al. [40] used titanium nitride (TiN) nanoparticles to incorporate into ternary Ni-W-P plating using pulse electrodeposition. The optimal concentration to obtain the best mechanical properties was reported to be 4 g/L TiN. At this concentration, the microhardness increased from 496.6 HV to 847.6 HV, the mean COF decreased from 0.663 to 0.325, and the corrosion rate decreased.

According to previous studies conducted by various researchers, EL Ni-W-P coatings are appropriate ternary coatings that can be used for incorporating nano-sized or hard ceramic particles because the incorporation of nanoparticles helps to enhance the surface characteristics or properties of the EL deposits. Among nanoparticles, TiO_2_ offers extensive applications owing to its unique properties, including improved hardness, corrosion rate, wear resistance, photocatalysis, water treatment, and environmental remediation. Nanoscale TiO_2_ exhibits high surface exposure, making it highly effective for applications that demand enhanced surface reactivity; thus, it is suitable for addressing various engineering challenges [41,42]. The TiO_2_ particles in composite coatings are used as an effective reinforcement, augment the strength and wear resistance of the composite, and provide good antibacterial properties with better photocatalytic and electrocatalytic properties [43,44]. Recently, Wang et al. prepared Ni-W-P-TiO_2_ nanocomposite coatings using a sol-based electroplating process. They recorded a microhardness of 580 HV with a 5 mL/L content of TiO_2_ sol. The compact and uniform layer of the deposits achieved with sol-TiO_2_ contributed to the optimal corrosion resistance, whereas abrasion wear played a key role in enhancing the wear resistance [45].

However, although significant studies have been conducted on electroplated Ni-W-P-TiO_2_ coatings, the incorporation of nano-TiO_2_ particles into ternary Ni-W-P via the electroless (EL) coating method remains relatively unexplored. Moreover, there is limited knowledge about how nano-TiO_2_ particles affect the roughness and structural integrity of EL ternary composites.

Thus, the present study examines the impact of TiO_2_ nanoparticle reinforcement in EL Ni-W-P coatings, paying particular attention to roughness and its correlation with mechanical characteristics, specifically microhardness and wear resistance. A detailed comparative analysis was performed between the as-plated ternary coatings and their nano-TiO_2_ composite counterparts, highlighting the impact of post-deposition annealing at 400 °C on the microstructural and functional characteristics of the composite deposits. The findings are discussed in the following sections.

## 2. Materials and Methods

### 2.1. Materials

#### 2.1.1. Substrate Preparation

API X60 pipeline steel was chosen as the substrate for the electroless coating, with each specimen precisely machined to flat coupons measuring 10 × 15 × 3 mm^3^ using wire-cut EDM. The detailed chemical composition of the substrate provided by the manufacturer Aesteiron Steels Pvt. Ltd., Mumbai, India. is presented in Table 1. Two slots were cut at the edges of the specimens to facilitate suspension from the hanging wire during coating.

#### 2.1.2. Chemicals and Reagents Used

All chemicals used in this study were of analytical grade and used without further purification. The specific reagents, their exact concentrations, and functional roles in the electroless deposition process are detailed in Table 2.

### 2.2. Synthesis of the Composite Coatings

The synthesis of ternary electroless Ni-W-P and composite Ni-W-P-nanoTiO_2_ composite coatings followed the procedure below, with all reagents listed in Table 2.

Before the deposition process, a series of pre-treatment procedures were implemented for cleaning and ensuring optimal coating adhesion. The cleaning process consists of three main steps:

- Mechanical substrate polishing was performed using emery papers with grit sizes ranging from coarse to fine (200 to 1500 GSM).
- Ultrasonic cleaning in acetone was performed for 10 min to effectively remove organic contaminants.
- Alkaline treatment was conducted using 10 wt.% sodium hydroxide at 50 °C for 5 min to eliminate residual dirt and foreign particles, in compliance with ASTM B 656 [46] standards.

Following each step, the substrate was thoroughly cleaned with deionized (DI) water to eliminate any residual cleaning agents before plating.

The EL bath was prepared by dissolving the required chemicals in 250 mL of deionized water under continuous stirring until homogeneity was achieved. The bath pH was adjusted and stabilized at pH 8 by using an ammonia solution to maintain the optimal basic conditions for deposition. The cleaned and rinsed pre-treated substrates were activated by immersion in a PdCl_2_ solution preheated to 55 °C for 12 s to promote a uniform distribution of catalytic sites, which improved substrate surface adhesion. Following activation, the samples were placed in an EL bath, where the deposition process was conducted at 85 ± 2 °C for one hour with constant stirring at 450–500 rpm using a REMI 5MLH (REMI Elektrotechnik Ltd., Mumbai, India) automatic hot plate cum magnetic stirrer. Initially, a Ni-W-P ternary layer was deposited for 20 min. Subsequently, TiO_2_ nanoparticles were introduced into the same bath, and the process was continued for another 40 min to obtain uniform Ni-W-P-nanoTiO_2_ composite deposits. After the deposition, the coating samples were removed and cleaned with DI water to eliminate any residual plating solution.

To improve the microstructural stability, strength, and tribo-mechanical performance of the annealing, the EL composite coatings were annealed or heat-treated (HT) at 400 °C in a tube furnace under a controlled temperature to avoid oxidation and ensure uniform thermal exposure. Thermal processing via annealing facilitated morphological refinement with improved mechanical properties, making these coatings suitable for tribological applications.

### 2.3. Characterization of the EL Coatings

#### 2.3.1. Optical Microscopy, SEM, EDAX, AFM, and XRD Test

The ternary and composite coatings, along with their cross-sectional thicknesses, were characterized using an upright optical microscope (Olympus BX53M, Tokyo, Japan) equipped with a digital camera and analysis software.

The surface morphology and microstructural features were examined using a field-emission scanning electron microscope (FESEM, Model: Sigma, Carl Zeiss Microscopy Ltd., Jena, Germany) coupled with an energy-dispersive X-ray spectroscopy system (EDAX, AMETEK, Berwyn, PA, USA) for elemental analysis. The FESEM was operated at an accelerating voltage of 20 kV in the high-vacuum mode, with a typical working distance of 8–10 mm. Before imaging, the samples were sputter-coated with a thin layer of gold to improve conductivity and resolution. Quantitative measurements of nodule size were performed using ImageJ software (v1.46) based on high-magnification FESEM micrographs.

Furthermore, the surface morphology at the nanoscale was investigated using an Atomic Force Microscope (NaioAFM, Nanosurf AG, Liestal, Switzerland) operating in the dynamic force (tapping) mode under ambient air conditions. A silicon cantilever (Dyn190AI, vibrational frequency ≈ 164 kHz) was used with a setpoint of 55% and a vibration amplitude of 60 mV. Scans were performed over an area of 15 × 15 µm^2^. Data acquisition and processing were performed using Nanosurf Control Software (v3.8.3.3), and feedback was managed using an adaptive PID control algorithm. Height profiles and surface roughness parameters were extracted from topographic data for comparative evaluation.

The phase and structural characterization of the electroless coatings were carried out using an X-ray diffractometer (Rigaku Miniflex-600, Tokyo, Japan), a benchtop instrument equipped with a Cu-Kα radiation source (λ = 1.54 Å). The measurements were conducted over a 2θ range of 20–75° at a scan rate of 1°/min, with a voltage of 40 kV and a current of 15 mA. The diffraction patterns were interpreted using Match3 software (v2.2.1) for phase identification. The average crystallite sizes of the coatings were estimated using the Debye–Scherrer equation, assuming negligible strain broadening and instrumental error correction.

#### 2.3.2. Adhesion Test

The adhesion between the electrolessly deposited coatings and steel substrates was examined using the cross-cut tape test under ASTM D3359-22 [47], Test Method B. Square specimens measuring 25 × 25 mm^2^, coated with Ni-W-P and Ni-W-P-TiO_2_, were used for the analysis. Because the coating thickness was <50 µm, 1 mm equidistant cuts were performed following the standard (ASTM D3359 [47]), forming a grid of squares, each approximately 1 mm^2^ in area. A cross-hatch cutter with 1 mm blade spacing was used to create the grid, and transparent scotch tape was employed for the adhesion test. The testing procedure and apparatus used are presented in the Supplementary Material (Figure S1). All tests were performed under ambient laboratory conditions (27 °C). Following the incisions, pressure-sensitive transparent adhesive tape was applied over the cross-hatched region and removed in a single swift motion. The coated areas were examined to assess the degree of material detachment. The percentage of coating removed was recorded as a measure of the adhesion quality. The test was conducted on both Ni-W-P- and Ni-W-P-TiO_2_-coated steel substrates, and each sample was tested three times for reproducibility.

#### 2.3.3. Surface Roughness Test

The areal surface roughness parameters (S_a_, in µm) of the coatings were measured using a Coherent Correlation Interferometer (CCI MP-L, Taylor Hobson, Leicester, UK) and a non-contact, high-resolution 3D optical profilometer based on white-light interferometry. The instrument offers sub-nanometer vertical resolution and is capable of capturing high-fidelity surface topography without physical contact with the sample. Measurements were performed under ambient conditions using a Gaussian filter following ISO 25178 [48] surface texture standards, with a cut-off length of 0.08 mm to suppress form and waviness components. The scan area was typically set to 0.9 mm × 0.9 mm, and data acquisition and surface analysis were conducted using Taylor Hobson’s analysis software (TalyMap, v6.4).

#### 2.3.4. Microhardness and Wear Test

Microhardness measurements were conducted using a UHL VMHT microhardness tester (UHL, Asslar, Germany) equipped with a Vickers diamond indenter operated with a dwell time of 12 s, an applied load of 50 gf, and an indentation speed of 25 μm·s^−1^. To account for surface heterogeneity and ensure measurement reproducibility, five indentations were made at randomly selected locations on each as-coated surface without further surface polishing. The reported Vickers microhardness values represent the arithmetic means and standard deviations of the measurements.

The tribological performance of the coatings under unlubricated conditions was evaluated using flat coupons of API X60 steel (Aesteiron Steels LLP, Mumbai, India) on a Universal Tribometer (Model: MFT-500, Rtec Instruments, San Jose, CA, USA) configured for a linear reciprocating motion. A 5 mm diameter 316 stainless steel (SS) ball was employed as the counterface material. The tests were carried out under a normal load of 5 N, stroke length of 5 mm, and sliding frequency of 3 Hz under ambient conditions (27 °C). Each test was performed for 300 s, corresponding to a total sliding distance of approximately 9 m. The coefficient of friction (COF) was continuously monitored to evaluate both the frictional stability and wear resistance. All tribological experiments were repeated thrice to ensure reproducibility, and the reported COF trends and wear track measurements represent the averaged values obtained from these independent trials.

## 3. Results

### 3.1. Morphology of the EL Coatings

#### 3.1.1. Optical Microscopy Study of EL Coatings

**Figure 1:**
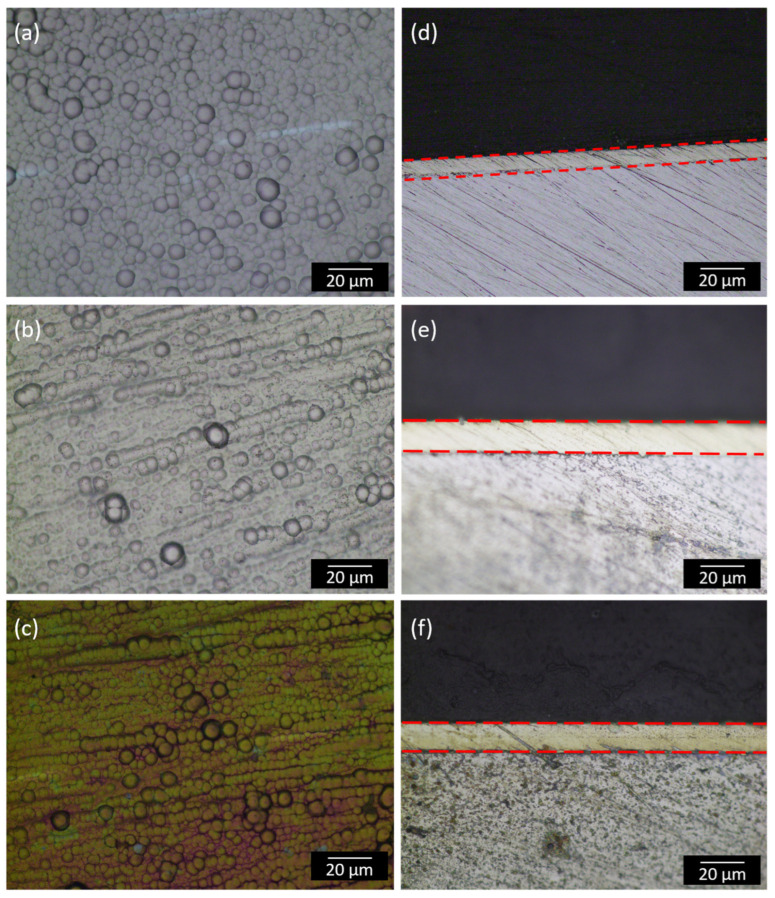
Optical microscopy images of the electroless deposits: (**a**,**d**) Ni-W-P and its cross-section, (**b**,**e**) as-plated Ni-W-P-nanoTiO_2_ and its cross-section, and (**c**,**e**) annealed Ni-W-P-nanoTiO_2_ and its cross-section, representing the coating thickness. (Red dotted lines in (**d**–**f**) represents the coating thickness).

Figure 1 shows the optical micrographs of the tungsten-based ternary alloy and Ni-W-P-nanoTiO_2_ EL composite films and highlights their structural characteristics. Ternary Ni-W-P exhibited a compact and uniformly distributed nodular morphology as shown in Figure 1a. The incorporation of TiO_2_ nanoparticles into Ni-W-P is evident in Figure 1b, where the composite coating exhibits a homogenous dispersion of nano-reinforcements, which is a characteristic feature of EL composite coatings and reflects the autocatalytic deposition process and uniform growth across the substrate. After annealing at 400 °C, the EL composite underwent a distinct color transformation to greenish-yellow, as shown in Figure 1c. The greenish, yellow, and purple hues visible in the optical micrograph may arise from the oxide layers [49] or from the precipitation of the Ni_3_P crystallite phase, which is often observed in thin electroless coatings [10].

Thickness analysis showed that the ternary tungsten-alloyed EL nickel deposits had a coating thickness of 20 µm, as shown in Figure 1d, while the Ni-W-P-nanoTiO_2_ composite deposits achieved a slightly increased thickness of nearly 24 µm, as depicted in Figure 1e,f, after one hour of deposition for both coatings. Notably, during the cross-sectional sample preparation for thickness measurements, the Ni-W-P deposits exhibited signs of delamination, suggesting weaker bonding to the substrate. Conversely, the Ni-W-P-nanoTiO_2_ deposits demonstrated significantly improved substrate bonding, remaining intact throughout the polishing process during the cross-sectional sample preparation for thickness measurements, and the ternary deposits showed delamination signs, indicating a weaker substrate adhesion. Conversely, the Ni-W-P-nanoTiO_2_ deposits exhibited considerably improved substrate adhesion and remained intact throughout the polishing process.

#### 3.1.2. SEM, EDAX, and XRD Study of the EL Coatings

**Figure 2:**
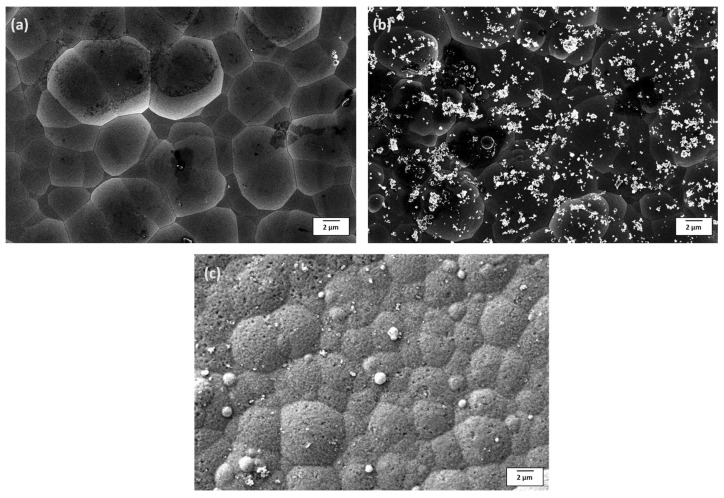
Morphology of the electroless deposits: (**a**) Ni-W-P, (**b**) Ni-W-P-nanoTiO_2_ as-plated, and (**c**) annealed Ni-W-P-nanoTiO_2_.

**Figure 3:**
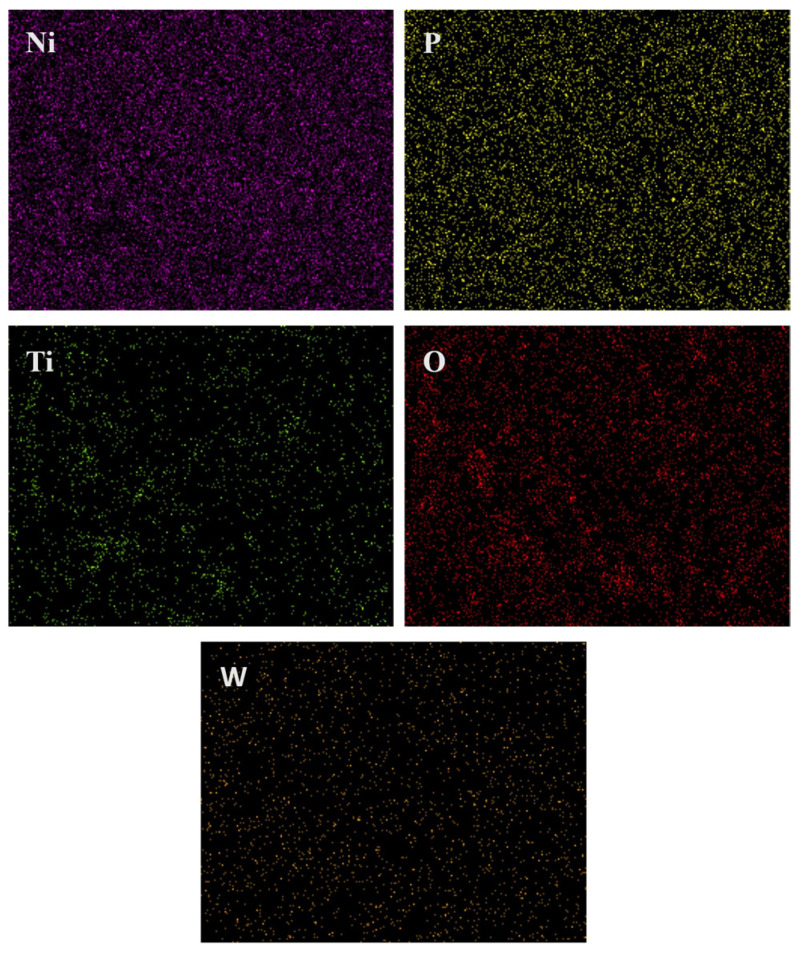
Elemental mapping of as-plated Ni-W-P-nanoTiO_2_.

Figure 2 shows the FESEM images illustrating the morphology of the as-plated EL tungsten alloy and Ni-W-P-nanoTiO_2_ composite films. Unlike the smooth surface of Ni-W-P, the co-deposition of TiO_2_ in the EL composite deposits resulted in a rough surface. The Ni-W-P coatings (Figure 2a) revealed a densely packed structure of spherical nodules that were uniformly distributed, with fewer observable defects such as pores, cracks, or cavities, ensuring their structural integrity and uniformity. The inclusion of TiO_2_ nanoparticles is evident in Figure 2b, where bright white particles are embedded within the nodular structure, confirming the successful reinforcement provided by the nanoparticles. The elemental mapping in Figure 3 reveals the presence of all the expected elements in the composite coatings, confirming successful co-deposition. Table 3 confirms the content of 5% titanium (Ti), indicating the incorporation of nano-TiO_2_ within the coating structure. Some agglomeration sites were identified within the coatings, likely resulting from the non-uniformity of the nanoparticle distribution in the alloy coating bath despite continuous stirring. The average nodule size for ternary EL coatings displayed in Figure 2a measures 6.1 ± 1.4 µm, while the Ni-W-P-nanoTiO_2_ deposits (Figure 2b) exhibit slightly smaller nodules with a diameter of approximately 5.6 ± 0.6 µm. These nodules appeared more homogeneous, reflecting the uniform distribution of TiO_2_ nanoparticles in the ternary alloy matrix. Electroless composite deposits heat-treated at 400 °C developed nanoporous spherical nodules, as shown in Figure 2c, with an increased size of 6.0 ± 0.4 µm. This growth is attributed to grain coarsening and phase transformation facilitated by annealing, which suggests that TiO_2_ nanoparticles initially inhibit nodular growth, but promote it after heat treatment at a particular temperature.

An increase in phosphorus (P) content from 4.1 wt.% in Ni-W-P films to 5.0 wt.% in Ni-W-P-nanoTiO_2_ deposits, accompanied by a reduction in tungsten (W) content, was observed in Table 3. The decrease in W is attributed to the dilution effect caused by TiO_2_ addition in the electroless bath [50], which affects the bath chemistry because of the adsorption between tungstate ions and TiO_2_ particles. Post-annealing at 400 °C revealed that the phosphorus content decreased, the nickel (Ni) content increased, and the titanium (Ti) content remained constant, as observed through EDAX (Table 3).

**Figure 4:**
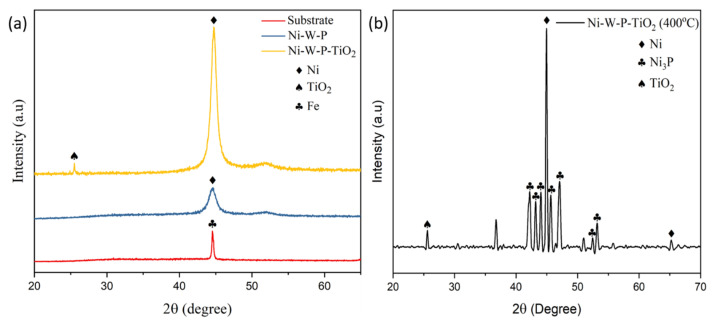
XRD spectra of electroless composite coatings: (**a**) substrate, Ni-W-P, and as-plated Ni-W-P-nanoTiO_2_, and (**b**) heat-treated at 400 °C Ni-W-P-nanoTiO_2_.

The XRD spectra of the substrate, ternary EL Ni-W-P, Ni-W-P-nanoTiO_2_, and annealed EL Ni-W-P-nanoTiO_2_ deposits are shown in Figure 4. The substrate exhibits only one peak at approximately 44.7° (2θ) and Fe (110). The EL Ni-W-P and Ni-W-P-nanoTiO_2_ composites exhibited an amorphous state, characterized by broad diffraction peaks at approximately 44.6° (2θ), corresponding to the face-centered cubic Ni (111) and (200) planes, with an estimated crystallite size of 5.76 nm. A minor peak at 25.02° (2θ), attributed to anatase TiO_2_ with the (011) plane, appears in both the as-deposited and annealed composite coatings. No distinct peaks for W and P were detected because these elements were dissolved within the Ni structure as a solid solution, preventing the development of separate phases [51,52]. The formation of microcrystalline Ni and crystallization of the Ni_3_P phase became evident after the heat treatment of the EL composites at 400 °C, as shown in Figure 4b. The crystalline structure of Ni_3_P was observed at 44.6° (2θ) (100), 47.1° (2θ) (101), 53.7° (2θ) (102), 56.7° (2θ) (110), and 68.4° (2θ) (110), consistent with previously reported results [53,54]. Following the annealed coatings, the crystallite size of the Ni (111) plane improved to 33.34 nm, which signifies a shift from amorphous to a more ordered crystalline arrangement, characterized by FCC Ni and body-centered tetragonal Ni_3_P. Additionally, the heat-treated composite coatings exhibited a strongly ordered orientation along the (111) and (200) planes. The EL composites annealed at 400 °C increased in grain size for the following reasons [55,56]: recrystallization, atomic diffusion and mobility, and phase transformation. This suggests that the incorporation of TiO_2_ nanoparticles promotes the alignment of Ni atoms along the (111) plane upon heat treatment. The different chemical compositions obtained from the EDAX data presented in Table 3 were in good agreement.

#### 3.1.3. Atomic Force Microscopy of EL Coatings

**Figure 5:**
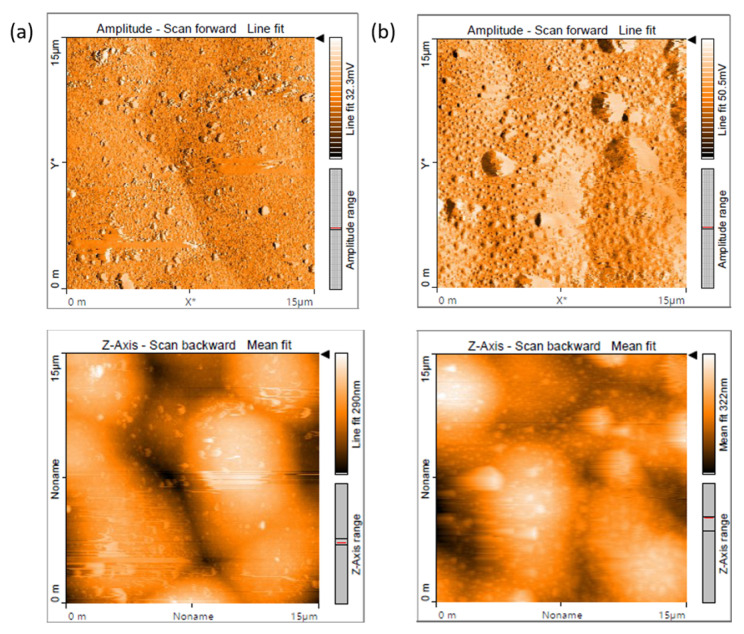
Amplitude and height scanned AFM images of (**a**) Ni-W-P and (**b**) Ni-W-P-nanoTiO_2_.

The topographies of the ternary and Ni-W-P-nanoTiO_2_ composite deposits were examined via Atomic Force Microscopy (AFM) with a scanning area of 15 × 15 µm^2^, as illustrated in Figure 5. The AFM results of the ternary coatings (Figure 5a) revealed a surface characterized by well-distributed nodule-like structures, a distinctive feature resulting from the autocatalytic mechanism of the electroless plating process. These densely packed nodules contribute to an even and compact morphology. Conversely, the AFM image of the composite coatings (Figure 5b) reveals that the coated surface was embedded with uniformly dispersed TiO_2_ nanoparticles. This uniform dispersion of nanoparticles indicates that TiO_2_ incorporation affects the texture of the ternary coatings and improves the tribo-mechanical characteristics of the EL Ni-W-P-nanoTiO_2_ deposits. The Z-axis AFM scan of the Ni-W-P coating showed a relatively smooth and uniform surface with a mean height variation of 290 nm, indicating compact nodular morphology and lower surface roughness. In contrast, the Ni-W-P-TiO_2_ composite exhibited a higher mean height of 322 nm with more pronounced topographical features, attributed to the inclusion of TiO_2_ nanoparticles. The “mean fit” captures overall surface variation, while the “line fit” highlights finer local features, together providing a comprehensive view of surface structure evolution.

### 3.2. Microhardness of the EL Coatings

The hardness of a material is a key property of a coating, as it indicates resistance to indentation and quantifies the ability of a material to withstand permanent deformation under a standard load [57]. The Vickers microhardness (HV) test is widely used because of its variability across a broad range of load applications. The HV of the material was determined by the Vickers hardness test using the formula

Vickers hardness=2 Fa Sin(θ2)d2

where *F*_a_ denotes the force applied in kilogram-force (kgf), d is the average diagonal (mm) of the indent, and θ is the diamond pyramid indenter’s opposite face angle, which is typically 136°.

**Figure 6:**
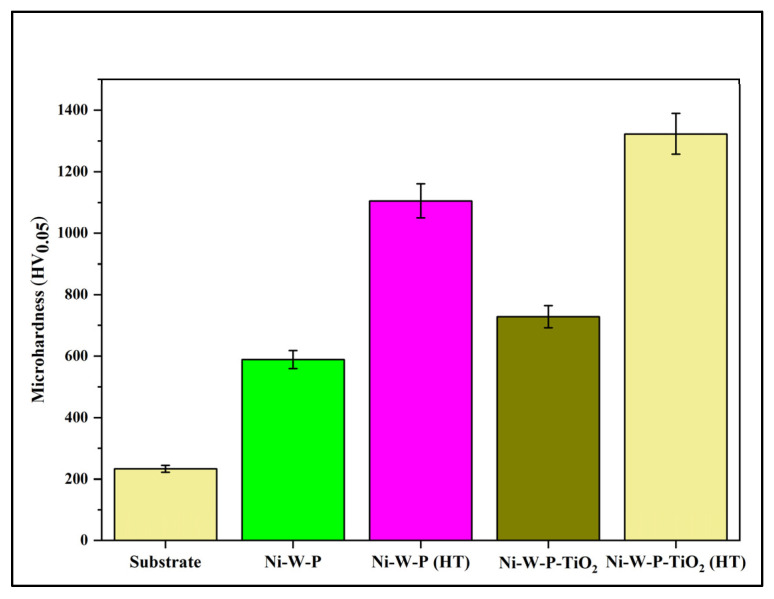
Microhardness comparison of the electroless deposits.

Table 4 and Figure 6 show the microhardness data obtained from the Vickers microhardness test for the X60 steel (substrate) along with the EL ternary and Ni-W-P-nanoTiO_2_ coatings, which were further heat-treated at 400 °C. The microhardness of API X60 steel was approximately 235 HV_0.05_.

Upon electroless deposition, Ni-W-P resulted in an increase in microhardness to 689 HV_0.05_, and with the inclusion of nano-TiO_2_ particles into the deposits, this value further improved to 728 HV_0.05_. Interestingly, heat treatment of the EL composite deposits at 400 °C further increased the microhardness to 1323 HV_0.05_. This increase in hardness is attributed to the presence of Ti, W, and a more stable nickel phosphide phase, as evidenced by the EDAX and XRD results. The increase in hardness could also be due to the embedded TiO_2_ nanoparticles, which were adsorbed onto the ternary alloy films owing to electrostatic attraction to the positively charged nickel ions. As the coating developed, these nanoparticles were entrapped within the ternary alloy films and consequently enhanced the microhardness of the coating. The observed increase in hardness was primarily attributed to three mechanisms: (i) the Orowan mechanism, (ii) grain refinement, and (iii) dispersion strengthening. The Orowan mechanism [58] hinders movement because dislocations must bypass hard TiO_2_ nanoparticles, which require additional energy, thus improving the resistance of the coating to plastic deformation. TiO_2_ nanoparticles also served as nucleation sites for grain formation, resulting in a finer grain structure. According to Hall–Petch’s relation, refinement of the grain structure increases the hardness by hindering dislocation movement [59]. Additionally, dispersed TiO_2_ nanoparticles, which are highly distributed in intergranular regions, can hinder grain coarsening and contribute to refinement and dispersion strengthening [58,59]. Therefore, it can be suggested that the presence of titania (TiO_2_, anatase crystalline phase evidenced by XRD) particles may hinder dislocation movement within the crystal lattice of the coating, thereby inhibiting plastic deformation and enhancing hardness.

### 3.3. Surface Roughness Study of the EL Coatings

**Figure 7:**
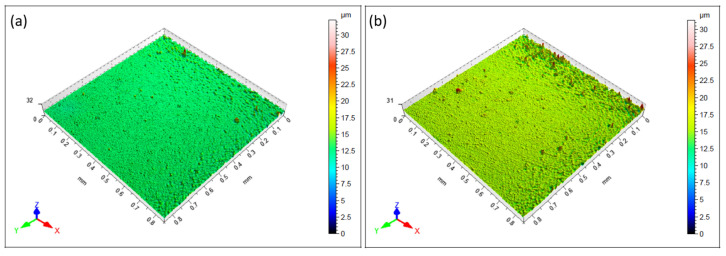
Three-dimensional roughness profiles of electroless (**a**) Ni-W-P and (**b**) Ni-W-P-nanoTiO_2_ deposits.

**Figure 8:**
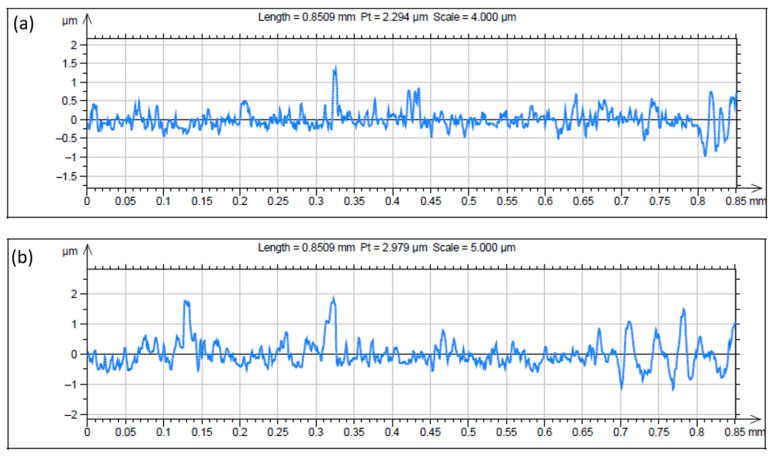
Two-dimensional roughness profiles of electroless (**a**) Ni-W-P and (**b**) Ni-W-P-nanoTiO_2_ deposits.

Figure 7 and Table 5 show the 3D profilometer surface profiles and roughness parameters of the ternary alloy and composite coatings containing 5 g/L nano-TiO_2_. The profilometry results in Figure 7a reveal a relatively smoother surface for the ternary coatings, with a peak-to-height (Pt) value of 2.294 µm, as shown in the profile plot, which is slightly less than that of the composite coatings, which is approximately 2.979 µm. The 3D map in Figure 7b indicates pronounced asperities, suggesting that the inclusion of nano-TiO_2_ increased the roughness, possibly because of agglomeration (Figure 2b). Figure 8 shows the 2D surface roughness profiles of ternary and nanoparticle-reinforced composite coatings. The ternary coating (Figure 8a) exhibits a smoother morphology with a peak-to-valley height (Pt) of 2.294 µm, whereas the incorporation of TiO_2_ nanoparticles (Figure 8b) leads to increased roughness (Pt = 2.979 µm). The rougher surface observed in the composite may be attributed to the agglomerated or heterogeneously distributed TiO_2_ particles embedded during deposition. The area roughness (S_a_) value obtained for the Ni-W-P coating was 0.2810 µm, and that for the composite coating was 0.4369 µm. The presence of Ti (as evidenced by EDAX analysis) in the composite coatings led to a higher S_a_ value. This suggests that the integration of nano-TiO_2_ particles within the ternary matrix increased the surface heterogeneity of the EL films [44] and altered the uniform structure of the Ni-W-P, creating topographical variations. Consequently, the composite coating exhibited an increased specific surface area compared with the smoother and more uniform ternary coatings. This finding is corroborated by the roughness measurements in Table 5, which indicate higher roughness values for the composite coatings. Despite its rough surface, the composite coating is expected to deliver superior performance in terms of load-carrying capacity and durability, particularly under harsh tribological conditions.

### 3.4. Adhesion Assessment of the Coatings

**Figure 9:**
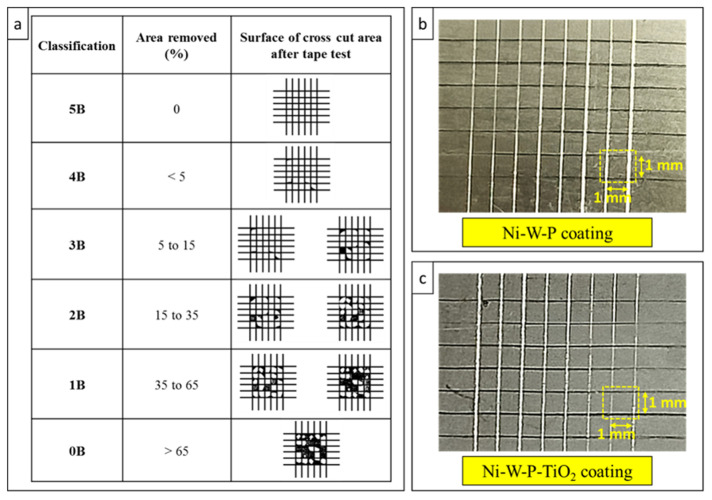
Adhesion test data. (**a**) Classification of adhesion strength according to the ASTM D3359-22 standard [47] for coated substrates after the tape test: (**b**) Ni-W-P and (**c**) Ni-W-P-TiO_2_.

For electroless plating to achieve reliable functional performance in practical applications, adequate interfacial adhesion between the deposited layer and substrate is a fundamental requirement. In this context, adhesion refers to the resistance against the separation of the coating from the substrate under mechanical stress. A qualitative assessment of the adhesion strength was performed using the cross-cut tape test following ASTM D3359-22 [47] Test Method B, as illustrated in Figure 9a. As shown in Figure 9b,c, the grid patterns remained largely intact following tape removal, with negligible detachment or flaking of the coating material. Based on the visual assessment criteria prescribed in the standard, both coating types demonstrated excellent adhesion, exhibiting a classification level of 5B, indicating no significant coating removal from the cross-cut area. The inclusion of TiO_2_ nanoparticles in the Ni-W-P matrix did not adversely affect adhesion; rather, the Ni-W-P-TiO_2_ coating maintained uniform adhesion across all tested specimens, confirming its mechanical compatibility with the steel substrate.

### 3.5. Wear Study of EL Coatings

**Figure 10:**
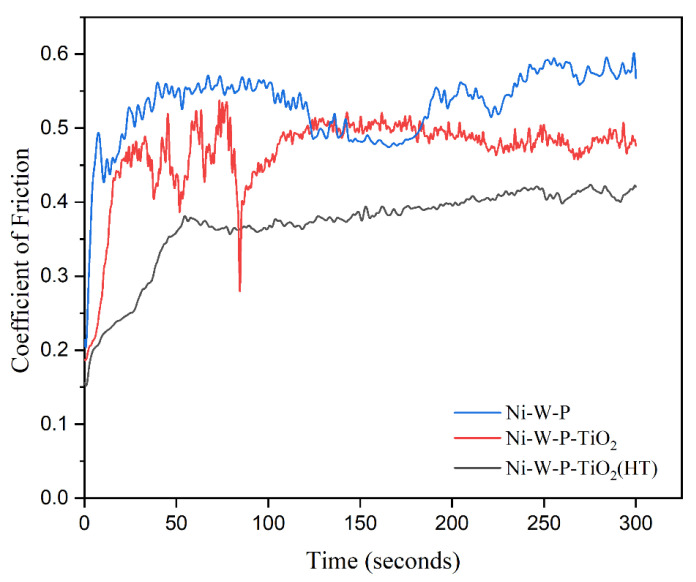
Influence of time on the COF of Ni-W-P and heat-treated and as-plated Ni-W-P-nanoTiO_2_ coatings.

Figure 10 illustrates the wear behavior of the EL ternary and as-plated annealed Ni-W-P-nanoTiO_2_ composites, showing the variation in the friction coefficient (COF) over time under a load of 5 N, with a steel ball as the counter body. The data demonstrated that the inclusion of nano-TiO_2_ nanoparticles into the ternary EL matrix and their subsequent heat treatment (400 °C) significantly reduced the COF relative to the nano-TiO_2_ composite and ternary coatings. The maximum COF, measured at 0.531, was associated with EL ternary Ni-W-P deposits, whereas a minimum COF of approximately 0.3691 was observed for the heat-treated nanoTiO_2_ composite coatings, underscoring the improved wear resistance imparted by TiO_2_ nanoparticles and annealing. The COF versus time profile of the ternary coating revealed a sharp initial increase during the early stages of the reciprocating wear test, followed by stabilization after approximately 50 s, reflecting the formation of a steady-state tribological interface. Furthermore, deterioration was observed between 120 and 200 s; meanwhile, the heat-treated and as-plated EL composite coatings showed a stable pattern after 100 s of the reciprocating wear test. The enhancement in resistance to wear is attributed to the combined effects of the robust interfacial bonding between the contact interfaces and the plowing action exerted by the hard, wear-resistant nano-TiO_2_ particles, which were homogeneously dispersed into the electroless nickel ternary film. Additionally, the integration of nano-TiO_2_ into the Ni-W-P film creates a “diffuse reinforcement effect,” hindering dislocation movement. This restriction reduces dislocation slip, enhances the deformation resistance of the material under stress, and improves the wear resistance [40].

The observed decrease in COF could be primarily due to the thermal annealing (at 400 °C) of the nano-TiO_2_ composite coatings, which concurrently facilitated the attainment of maximum hardness, as shown in Table 4. This improvement was primarily driven by the integration of titania and tungstate within the EL ternary coatings, as confirmed by EDAX (see Table 3). Moreover, the thermal treatment induced the crystallization of hard Ni_3_P, which likely played an influential role in augmenting the hardness and wear performance of the electroless composite deposits [13,61]. These findings are in good agreement with the microhardness data.

**Figure 11:**
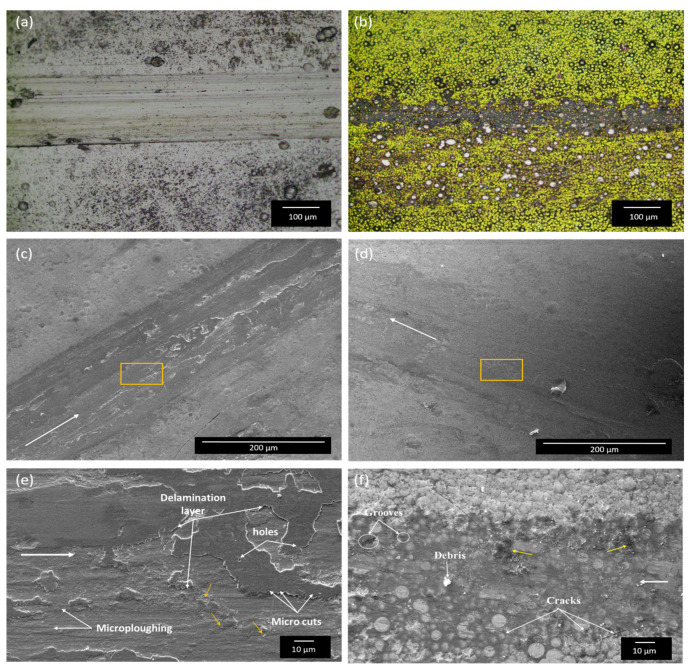
Microscopic and SEM morphologies of the worn surfaces of the electroless coatings: (**a**,**c**,**e**) as-plated Ni-W-P-nanoTiO_2_ and (**b**,**d**,**f**) Ni-W-P-nanoTiO_2_ heat treated at 400 °C.

Optical microscopy and SEM were used to examine the underlying mechanism of wear that governs the tribological characteristics of the EL deposits. The SEM images of the worn-out coated samples for both the heat-treated (annealed) coatings and as-plated EL deposits after 300 s of reciprocating wear testing are illustrated in Figure 11. The microscopic images in Figure 11b reveal that the worn-out track length of the nano-TiO_2_ composite coatings annealed at 400 °C is shorter than that of the as-plated EL deposits, which exhibit wider worn-out tracks with pronounced plow lines (Figure 11a) under the same testing load of 5 N. A wider and deeper track length is a direct measure of wear volume loss [62]. This disparity is likely caused by the lower microhardness of the EL composites in contrast to the annealed composite coatings. The reduced plowing depth and narrower wear track observed in the annealed EL Ni-W-P-nanoTiO_2_ composite deposits underscore their superior wear performance. This enhancement in wear performance is due to the annealing of the composite coatings at a temperature of 400 °C, which promotes structural densification, which increases the nano-TiO_2_ composite coating hardness and the reinforcement provided by TiO_2_ nanoparticles. The nano-TiO_2_ particles improved the load-bearing capability and effectively mitigated material deformation during reciprocating wear, thereby improving the overall tribological performance.

The wear track of the as-deposited EL coatings (Figure 11a) exhibited a dark gray to black appearance, which resulted from material transfer, oxidation, or microstructural changes in the wear track owing to heat and friction during reciprocating wear. The yellow arrows in Figure 11 indicate the presence of the wear debris. Furthermore, the wear scars and scratches were aligned in the same direction as the sliding motion, with distinct deep grooves and flattened patches, which are characteristic of an abrasive wear mechanism [63,64]. The worn surfaces also exhibit wide grooves with poor adhesion, which is likely attributed to surface oxidation, plastic deformation, and severe counterpart wear caused by the incorporation of nano-TiO_2_. Makkar et al. [65] identified similar behavior of the adhesive mechanism in Ni-P-TiO_2_ coatings at basic pH. However, TiO_2_ nanoparticles can serve as solid lubricants, enhancing wear performance during reciprocating wear [66,67]. The yellow rectangle on the worn track highlighted in Figure 11c,d shows the region where the EDAX analysis of the coatings was conducted. The EDAX data presented in Table 6 reveal the presence of a very low amount of Fe, which is believed to originate from the ferrous counterpart during tribo-testing, supporting the high Ni content of the wear track.

Figure 11 b,d,f shows a few abrasive grooves or micro-cuts, globules, or nodules that are visible after the wear test under the same load conditions. With an increase in time, they were flattened upon the tribo-test, and a few black sites (shown by the yellow arrow in Figure 11f) were observed, suggesting oxide formation. This indicates that after annealing at 400 °C, the composite coating became more resistant to wear because of the increased hardness owing to the presence of W content with better-distributed or dispersed TiO_2_ nanoparticles and phase-transformed hard Ni_3_P (see the XRD data in Figure 4b) available in the composite matrix. This observation indicates a synergistic effect of W and TiO_2_, where W contributes to the hard phases in the matrix, whereas TiO_2_ serves as a lubricious property. However, the morphology (Figure 11f) of the heat-treated composite coatings showed some cracks at the edge of the worn surfaces because of the high hardness, owing to precipitation hardening, leading to brittleness in the composite coatings. Consequently, the wear mechanism in the EL nano-TiO_2_ composites can be identified as abrasive wear, instigated by the co-deposition of nano-TiO_2_. Furthermore, signs of adhesive wear were observed, which likely contributed to the improved wear resistance of the EL composite.

## 4. Conclusions

This study shows that integrating nano-TiO_2_ into ternary Ni-W-P coatings through an alkaline hypophosphite-reduced electroless plating process can substantially enhance the mechanical and tribological performance required for pipeline protection. The coatings demonstrated excellent adhesion to steel substrates, achieving a top 5B rating in cross-cut tests without any visible detachment, which is crucial for maintaining long-term integrity under pipeline operating conditions. Heat treatment induced a transformation from the amorphous to crystalline Ni_3_P phase, improving the structural stability and increasing the hardness to approximately 1323 HV0.05. The process produced uniform, defect-free coatings up to 24 µm thick, while the addition of TiO_2_ nanoparticles increased the surface roughness, helping the coatings to better withstand abrasive flow and mechanical contact inside pipelines. The combined effect of hard tungsten, dispersed nano-TiO_2_ particles, and crystalline Ni_3_P greatly improved the wear resistance, particularly after annealing, with adhesive and abrasive wear identified as the dominant mechanisms.

These findings highlight the potential of nano-TiO_2_-reinforced Ni-W-P electroless composite coatings to enhance the durability of pipelines in harsh environments. Future research could focus on optimizing nanoparticle content, exploring alternative ceramic or hybrid reinforcements, conducting economic analysis for large-scale feasibility, and evaluating performance under cyclic loading and corrosive conditions to broaden this coatings industrial applicability.

## Figures and Tables

**Table 1:** Chemical composition of API X60 pipeline steel (wt.%).

Element	C	Mn	P	Si	Cr	Ni	Mo	Cu	Ti	Fe
Weight%	0.068	1.396	0.015	0.206	0.021	0.016	0.001	0.009	0.018	Balance

**Table 2:** List of chemicals utilized with their concentrations and specific roles in the electroless deposition process.

Chemical Name	Supplier	Concentration (g·L^−1^)	Role
Nickel sulfate hexahydrate	Merck, Mumbai, India	35	Nickel ion source
Sodium tungstate dihydrate	Merck, Mumbai, India	10	Tungsten ion source
Sodium hypophosphite monohydrate	Merck, Mumbai, India	18	Reducing agent
Tri-Sodium citrate dihydrate	Merck, Mumbai, India	10	Primary complexing agent
Citric acid	Merck, Mumbai, India	5	Secondary complexing agent
Sodium dodecyl sulfate (SDS)	Merck, Mumbai, India	1 × CMC = 2.393 g·L^−1^	Surfactant
Titanium dioxide ultrapure nanopowder	SRL Pvt. Ltd., Mumbai, India.	5	Reinforcement agent
Palladium chloride	Merck, Mumbai, India	Used in activation	Substrate surface activation
Ammonia solution	Merck, Mumbai, India	-	Maintains the pH of the bath

Fresh solutions were prepared immediately prior to use to ensure consistency.

**Table 3:** Elemental analysis (determined by EDAX) of the EL ternary and composite coatings.

Element	Ni-W-P		Ni-W-P-NanoTiO_2_		Ni-W-P-NanoTiO_2_ (400 °C)	
	Weight %	Atomic %	Weight %	Atomic %	Weight %	Atomic %
P	4.1	7.8	5.0	8.5	2.82	4.56
Ni	89.7	90.2	85.3	76.9	87.81	74.86
Ti	-	-	1.9	2.1	2.0	2.09
O	-	-	3.4	11.2	5.77	18.06
W	6.2	2.0	4.4	1.3	1.60	0.44

**Table 4:** Vickers indentation microhardness results of the EL Ni-W-P and Ni-W-P-nanoTiO_2_ coatings.

Coatings	Vickers Microhardness (HV_0.05_)	
	As-Plated	Annealed [Heat Treated (HT) at 400 °C]
Ni-W-P	589 ± 20	1105 ± 33
Ni-W-P-nanoTiO_2_	728 ± 198	1323 ± 42

**Table 5:** Three-dimensional profilometer surface roughness parameter values.

Coatings	Height Parameters (ISO 25178 [48])				Amplitude Parameters-Roughness Profile (ISO 4287 [60])	
	S_a_ (µm)	S_q_ (µm)	S_ku_	S_z_ (µm)	R_a_ (µm)	R_q_ (µm)
Ni-W-P	0.2810	0.5719	122.1	32.26	0.1480	0.5452
Ni-W-P-nanoTiO_2_	0.4369	0.8395	45.59	31.36	0.2467	0.8244

**Table 6:** EDAX of the wear track shown by the yellow box in Figure 11c,d.

Coatings	Track Length	Ni	W	P	Ti	O	Fe
Ni-W-P-nanoTiO_2_ (400 °C)	199 µm	74.47	0.23	2.07	0.07	22.97	0.28
Ni-W-P-nanoTiO_2_	346 µm	73.70	0.43	2.08	0.40	23.09	0.47

## Data Availability

The original contributions of this study are included in this article and Supplementary Materials. Further inquiries can be directed to the corresponding author.

## Supplementary Materials

The following supporting information can be downloaded at https://www.mdpi.com/article/10.3390/ma18173949/s1: Figure S1: (a) Instruments used for the cross-cut adhesion test; (b) Schematic illustration of the test procedure, performed according to ASTM D3359 standard.
