# Supplementary material for: Nanoparticle-Reinforced Electroless Composite Coatings for Pipeline Steel: Synthesis and Characterization
Source: Materials (Basel). 2025 Aug 22;18(17):3949. doi: 10.3390/ma18173949 (PMC12429091; doi:10.3390/ma18173949)
Supplement: Supplementary file 1 [file materials-18-03949-s001.zip › materials-3777930-supplementary.pdf]

Supplementary Materials

The adhesion test procedure follows creating the equidistant parallel cuts (Figure S1b) using the apparatus depicted in Figure S1a. For the cuts, the instrument (A)—a cross-hatch cutter equipped with a multi-blade cutting tool (B)—was used to make a grid pattern of X and Y-direction incisions, as per ASTM D3359 guidelines. The cutter was pressed firmly and drawn steadily across the coated surface to produce a series of uniform, equally spaced cuts. The sample was then rotated 90°, and the same process was repeated to form the cross-cut grid. Following this, a standardized pressure-sensitive adhesive tape (C) was applied over the cross-hatched area. The tape was pressed firmly to ensure consistent contact and then removed at an angle close to 180° with a single, smooth motion. The magnifying lens (D) was employed to visually assess coating removal and classify the adhesion performance based on the standard rating scale. The hex key (E) was used for tightening or adjusting the cutter blades before each test to ensure consistent depth and sharpness of incision. All tests were conducted under ambient laboratory conditions (27 °C), and the entire procedure is illustrated in Supplementary Figure S1.

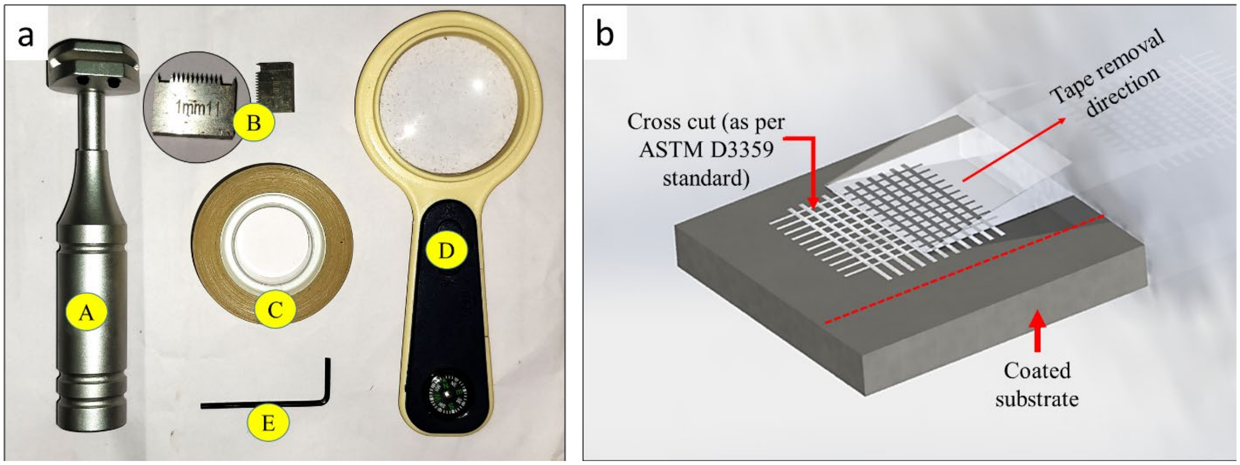

Figure S1. (**a**) Instruments used for the cross-cut adhesion test; (**b**) Schematic illustration of the test procedure, performed according to ASTM D3359 standard.
